# Isothermal compressibility and isobaric thermal shrinkage of a porous α-alumina compact: thermodynamic calculations

**DOI:** 10.3906/kim-2003-16

**Published:** 2020-06-01

**Authors:** Yüksel SARIKAYA, Müşerref ÖNAL, Abdullah Devrim PEKDEMIR

**Affiliations:** 1 Department of Chemistry, Faculty of Science, Ankara University, Ankara Turkey; 2 Graduate School of Natural and Applied Sciences, Ankara University, Ankara Turkey

**Keywords:** Activation energy, α-alumina, compressibility, shrinkage, thermodynamics

## Abstract

Two methods were proposed to calculate the thermodynamic parameters of porous ceramic compacts depending on their molar volume change with applied pressure and heating temperature, respectively. Molar volume of the porous α-alumina (α-Al_2_O_3_) compact was evaluated according to literature depending on both the applied pressure at room temperature and the heating temperature at atmospheric pressure. The isothermal compressibility coefficient, Gibbs energy, and work done on the compact by compression were calculated. The thermal shrinkage coefficient and activation energy as well as the change in enthalpy, entropy and Gibbs energy were calculated for partial sintering. The spontaneous nature of the treatments were discussed with respect to the obtained results.

## 1. Introduction

Several low temperature phases of aluminum oxide convert to α-alumina (α-Al_2_O_3_) through heating over 1150 °C [1,2]. Its natural form is known as the corundum mineral. Because of its high mechanical, thermal, and chemical stability porous α-alumina ceramics have been used in different areas such as catalyst support, membrane, electrode and thermal insulator. [3–5]. The void volume in a compact solids with widths smaller than 2 nm, between 2–50 nm and larger than 50 nm are called micropore, mesopore, and macropore, respectively [6,7].

The change in the relative volume of a porous compact with increasing pressure at constant temperature is defined as the isothermal compressibility [8–10]. Similarly, change in the relative volume of a porous compact with increasing heating temperature at constant pressure is defined as the isobaric thermal shrinkage [11,12]. The driving force for shrinkage is the chemical potential (molar Gibbs energy) difference of the mobile chemical species between the active center on the surface of the meso- and micropores in the compact [13,14]. Shrinkage occurs via the transfer of these species from higher to lower chemical potentials through several mechanisms such as grain boundary, surface and volume diffusions as well as evaporation and condensation [15–17].

There are numerous experimental results found in the literature for the kinetics and thermodynamics of isothermal compressibility [18–20], thermal shrinkage [21–23], and sintering [24–27]. Although there have been several mathematical modelling or computer simulations attempts, there are no studies concentrated on numerical calculations using basic thermodynamic relations. So, the aim of the present study is to calculate the change in some thermodynamic quantities during the isothermal compressibility and isobaric thermal shrinkage of a porous α-Al_2_O_3_ compact using the data evaluated from literature.

## 2. Materials and methods

The molar volume of the calcined porous α-Al_2_O_3_ compact was derived from literature depending on the applied pressure at constant temperature, as well as the heating temperature at constant pressure [6]. In addition, the isothermal compressibility and thermal shrinkage coefficients, together with the change in some thermodynamic quantities such as work, enthalpy, entropy and Gibbs energy were calculated, respectively. All results were given in SI units.

## 3. Results and discussion

### 3.1. Isothermal compressibility

**Figure 1:**
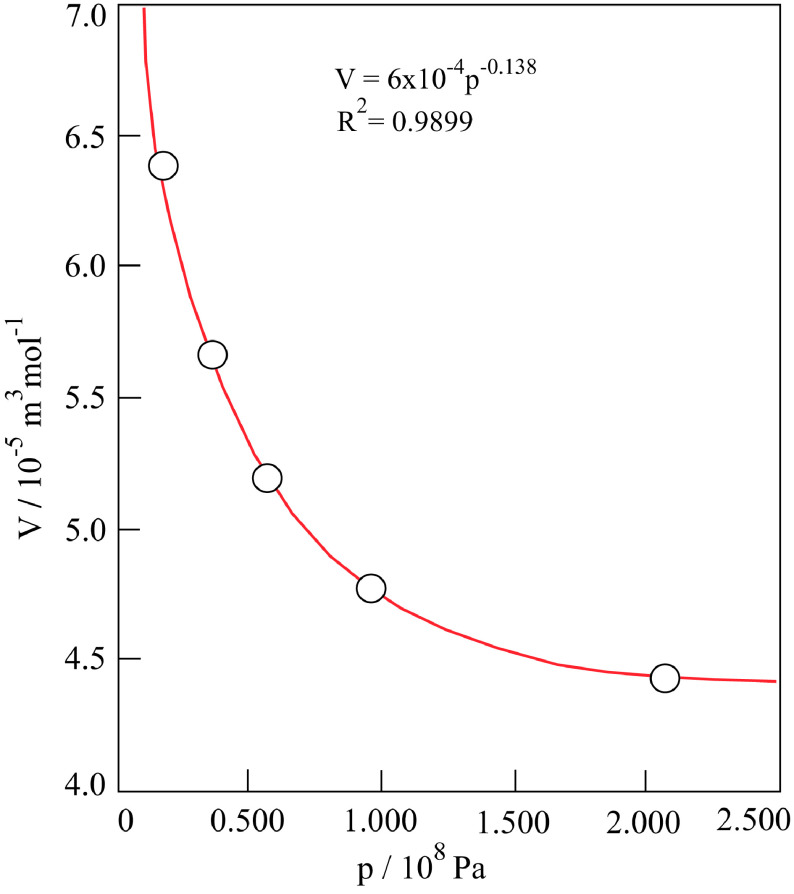
Variation in molar volume of α-Al_2_O_3_ compact with applied pressure.

The molar volume (V ) of the cylindrical porous α-Al_2_O_3_ compacts calcined at 1200 °C for 2 h were calculated using their diameter and thickness taken from the literature data depending on the applied pressure (p) ranging from 140 to 2080 bar (Table 1). The plot of V against p shows a decreasing curve in Figure 1. This change is due to the reduction of macropores to mesopores through application of extremely high pressing pressure on the compact at constant temperature whereas the meso- and micropores remain unchanged. The isothermal compressibility coefficient (κ) can be derived from the power equation of this curve given below;

(1)V=6x10-4/p0,138

(2)κ=-1V(∂V∂p)T=0,138p

where the negative sign has been incorporated into the definition so that κ is positive. The κ values produce a linear change with 1/p calculated for each pressure (Table 1).

**Table 1:** Pressing pressure (p) , molar volume (V ) and isothermal compressibility (κ) for a porous α-Al_2_O_3_ compact after calcination at 1200 °C for 2 h.

*p* /10^8^Pa	0.140	0.350	0.555	0.970	2.080
*V* /10^-5^ m^3^mol^-1^	6.390	5.671	5.205	4.781	4.451
*κ* /10^-9^ Pa^-1^	9.857	3.943	2.486	1.423	0.663

The Gibbs energy (ΔG) for the isothermal compression was calculated between the pressures *p_1_* = 1.40 × 10^7^ Pa and *p_2_* = 20.80 × 10^7^ Pa from the well-known basic thermodynamic relation:

(3)(∂G∂p)T=V

(4)ΔG=∫p1p2Vdp

(5)ΔG=∫p1p2(6x10-4/p0.138)dp=9293Jmol-1

The positive value of ΔG is due to the unspontaneous nature of the compression.

It has been internationally accepted that the work done on a system is positive and from the system is negative. Mechanical work done on the compact during the compression process has been calculated using the d(pV) = pdV + Vdp relationship in the following form;

(6)δw = − pdV = −d(pV) + Vdp

(7)w=-∫V1∫V2pdV=-∫p1V1p2V2d(pV)+∫p1p2Vdp

(8)=-p2V2+p1V1+ΔG

=(-2080x4.451+140x6.39)Jmol-1+9293Jmol-1=930Jmol-1

where the unit for pressure and volume is N/m^2^ and m^3^/mol, respectively. The inequality of ΔG /= w indicates irreversibility of isothermal compression according to the second law of thermodynamics.

### 3.2. Thermal shrinkage

**Figure 2:**
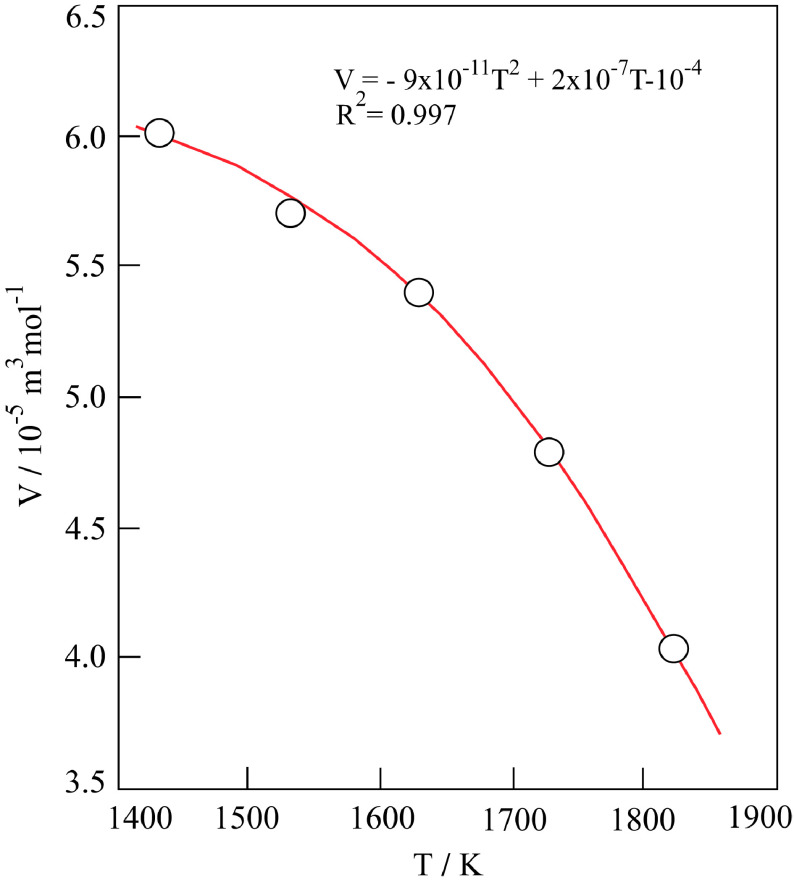
Variation in molar volume of α-Al_2_O_3_ compact with absolute sintering temperature.

Molar volume (V ) of the cylindrical porous α-Al_2_O_3_ compacts compressed at 280 bar was calculated using the diameter and thickness taken from literature data, for temperatures (T) in the range of 1150–1550 °C (Table 2). The plot of V against T reveals a decreasing curve in Figure 2. This change originates from the reduction of mesopores to micropores, followed by the closing of these micropores with increasing heating temperature at constant pressure. On the other hand, the macropores remain almost unchanged. The polynomial equation of this curve was evaluated as given below:

(9)V=-9x10-11T2+2x10-7T-10-4

where T is the absolute temperature.

**Table 2:** Heating temperature (T), molar volume (V) and thermal shrinkage (α) for a porous α-Al_2_O_3_ compact.

t/°C	1150	1250	1350	1450	1550
T/K	1423	1523	1623	1723	1823
V/10^-5^m^3^mol^-1^	6.00	5.69	5.41	4.79	4.04
−(∂V_T_/∂T)_p_/10^-8^m^3^mol^-1^K^-1^	5.614	7.414	9.214	11.014	12.814
α/10^-4^K^-1^	9.36	13.03	17.03	22.99	31.72
-ln α	6.974	6.643	6.375	6.075	5.754
T^-1^/10^-4^K^-1^	7.027	6.566	6.161	5.804	5.485

**Figure 3:**
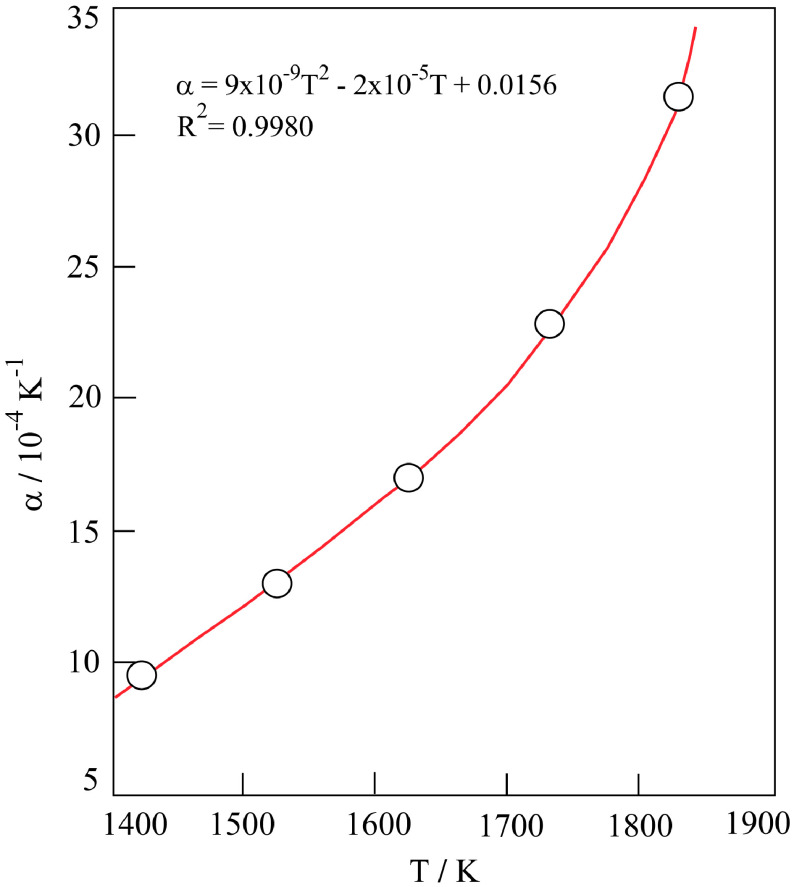
Variation in thermal shrinkage coefficient with absolute sintering temperature.

The thermal shrinkage coefficient (α) was calculated for each temperature using the following definition;

(10)α=-1V(∂V∂T)P

and given in Table 2. The curvilinear change of α versus T is depicted in Figure 3.

**Figure 4:**
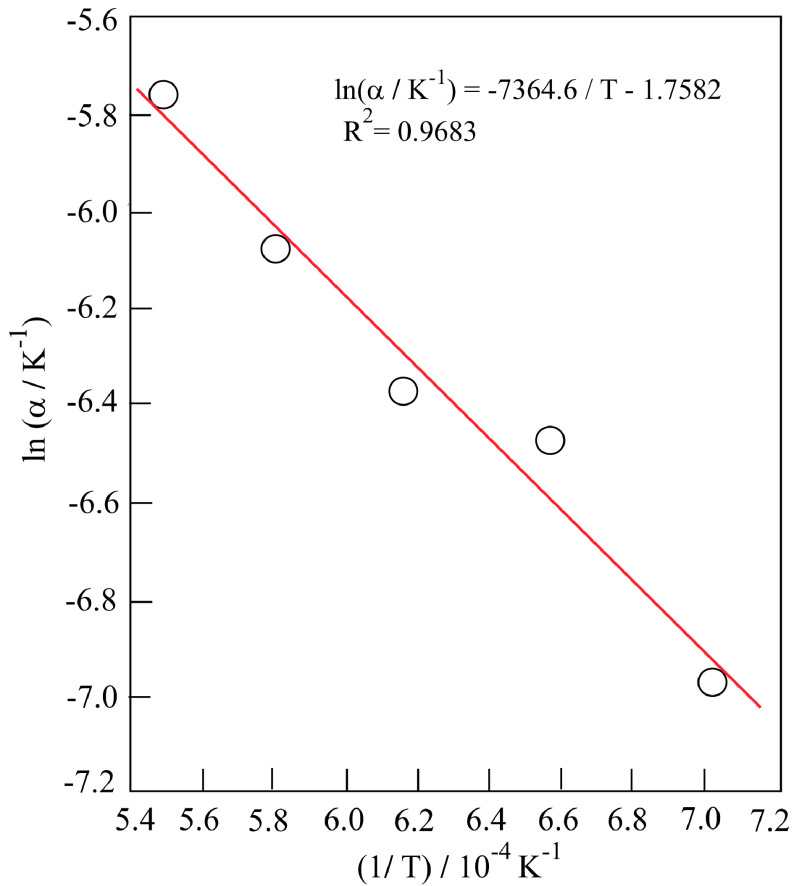
Arrhenius plot for the thermal shrinkage.

Since the plot of ln α against 1/T shows a linear relationship as seen in Figure 4, α behaves like a reaction rate constant and obeys the well-known Arrhenius equation;

(11)lnα=lnA-E#RT

where A is the preexponential factor, E^#^ is the activation energy, R is the universal gas constant, and T is the absolute temperature. The Arrhenius relation for thermal shrinkage was evaluated according to equation of the linear change shown in Figure 4:

(12)lnα = −17582-7364.6T

By comparison of the last two relations, the apparent activation energy for the thermal shrinkage can be calculated as follows:

(13)E#=(slope)R(13)

E#=(7364.6K)x(8.314JJK-1mol-1)=61229Jmol-1

The enthalpy and entropy changes (ΔH and ΔS) of the α-Al_2_O_3_ compacts during shrinkage between the temperatures T_1_ = 1423 K and T_2_ = 1823 K were calculated using the basic thermodynamic relations as given below:

(14)(∂H/∂T)p=Cp

(15)ΔH =∫T1T2CpdT=Cp(T2T1)

ΔH=(79.04JK-1mol-1)x(1823-1423)K=31616Jmol-1

and

(16)(∂S/∂T)p=Cp/T

(17)ΔS =∫T1T2CpTdT=CplnT2T1

ΔS=(79.04JK-1mol-1)ln18231423=19.6JK-1mol-1

where C_p_ (79.04 J K^-1^mol^-1^) is the temperature independent heat capacity of the α-Al_2_O_3_ [28]. The positive values originate from the increasing enthalpy and entropy of the compacts by during the thermal shrinkage. The inequality of ΔH<E^#^ shows the endothermic nature of thermal shrinkage which is partly sintering.

The temperature dependency of the Gibbs energy (ΔG) was also calculated using the last two results in via the basic thermodynamic relation given below:

(18)ΔG=ΔH-TΔS

(19)ΔG=31616-19.6T

Since thermodynamic equilibrium is established at ΔG = 0, the equilibrium temperature can be obtained as follows:

(20)T=ΔH/ΔS

T=31616Jmol-1/19.6JK-1mol-1=1613K(1340C)

Therefore, the value of ΔG is positive before 1340 °C and negative afterwards. The case where ΔG>0 that reveals thermal shrinkage is an unspontaneous process. It is spontaneous when ΔG<0. Finally, temperature dependency of the equilibrium constant (K) for the partly sinterization of the porous α-alumina compact can evaluate using the van’t Hoff equation.

(21)lnK=-ΔGRT=-31616+19.6TRT=-3803T+19.6

## 4. Conclusion

Some thermodynamic interpretations have proposed on the isothermal compressibility and isobaric thermal shrinkage of a porous ceramic compact. Change in the molar volume of a porous solid compact with pressure at constant temperature and with the temperature at constant pressure has been experimentally determined. Isothermal compressibility and thermal shrinkage of the compact were theoretically examined using the corresponding molar volume change with the basic kinetic and thermodynamic relationships. The unspontaneous nature and irreversibility of these processes were also discussed depending on the result of the thermodynamic calculations. The activation energy for the partial sintering of the porous compacts during thermal shrinkage was determined using the thermal shrinkage coefficient in the Arrhenius equation. In addition, sintering stages can distinguish from each other depending on different activation energies.
